# Retraction Note: Combination of sphingosine-1-phosphate receptor 1 (S1PR1) agonist and antiviral drug: a potential therapy against pathogenic influenza virus

**DOI:** 10.1038/s41598-023-34694-8

**Published:** 2023-05-10

**Authors:** Jiangnan Zhao, Meiying Zhu, Hao Jiang, Simen Shen, Xin Su, Yi Shi

**Affiliations:** 1 Department of Respiratory and Critical Medicine, Jinling Hospital, Nanjing University School of Medicine, Nanjing, 210002 China; 2 Department of Emergency Medicine, the Second Affiliated Hospital, Southeast University, Nanjing, 210002 China; 3 Department of Respiratory Medicine, the First People’s Hospital of Nantong, Nantong, 226000 China

Retraction of: *Scientific Reports* https://doi.org/10.1038/s41598-019-41760-7, published online 27 March 2019

The Editors have retracted this Article.

After publication, it was brought to the Editors' attention that the S1PR1 ECKO middle panel in Figure 2C appears to partially overlap with S1PR1 ECKO + Vehicle in Figure 3D in^1^. Further checks by the publisher found additional cases of potential overlap between western blots presented in Figure 1A (S1PR1 Lung VEH and S1PR1 Liver VEH, flipped horizontally), as well as between Figure 4A (p-JNK1/2 lanes 1 and 2) and B (p-JNK1/2 lanes 2 and 1, flipped horizontally).

The Editors requested the original raw data to address these concerns, but the Authors were not able to provide the full data, nor did the data provided contain sufficiently detailed metadata to allow for verification. The Editors therefore no longer have confidence in the reliability of the data presented in this Article.

None of the Authors have responded to correspondence from the Editors about this retraction.
